# The Role of Hydrogen Sulfide Targeting Autophagy in the Pathological Processes of the Nervous System

**DOI:** 10.3390/metabo12090879

**Published:** 2022-09-17

**Authors:** Huijie Zhao, Yihan Yang, Huiyang Liu, Honggang Wang

**Affiliations:** 1 Institute of Chronic Disease Risks Assessment, Henan University, Jinming Avenue, Kaifeng 475004, China; 2 School of Basic Medical Sciences, Henan University, Kaifeng 475004, China

**Keywords:** autophagy, cognitive impairment, diabetes depression, hydrogen sulfide, Parkinson’s disease, traumatic brain injury

## Abstract

Autophagy is an important cellular process, involving the transportation of cytoplasmic contents in the double membrane vesicles to lysosomes for degradation. Autophagy disorder contributes to many diseases, such as immune dysfunction, cancers and nervous system diseases. Hydrogen sulfide (H_2_S) is a volatile and toxic gas with a rotten egg odor. For a long time, it was considered as an environmental pollution gas. In recent years, H_2_S is regarded as the third most important gas signal molecule after NO and CO. H_2_S has a variety of biological functions and can play an important role in a variety of physiological and pathological processes. Increasingly more evidences show that H_2_S can regulate autophagy to play a protective role in the nervous system, but the mechanism is not fully understood. In this review, we summarize the recent literatures on the role of H_2_S in the pathological process of the nervous system by regulating autophagy, and analyze the mechanism in detail, hoping to provide the reference for future related research.

## 1. Introduction

Autophagy refers to a complex molecular pathway in which intracellular components are transported to the lysosome chamber for degradation and recycling [1,2]. According to the different ways in which substrates enter lysosomes, there are three types of autophagy: macroautophagy, microautophagy and chaperone-mediated autophagy [3]. As an evolutionarily conservative process, autophagy helps cells adapt to various stress conditions by providing amino acid libraries through the decomposition of proteins and peptides. Therefore, autophagy maintains intracellular homeostasis, enabling cells to survive [4]. Autophagy dysfunction is associated with many diseases, such as cancer, metabolic diseases, neurodegenerative diseases and lung diseases [3,5]. 

Hydrogen sulfide (H_2_S) is a volatile, flammable and toxic gas with a rotten egg smell, which can be detected by the human nose at a very low content [6]. Recently, it has been regarded as a biological signal molecule together with nitric oxide (NO) and carbon monoxide (CO) [7,8]. H_2_S is involved in many physiological and pathological processes in the body, such as ischemia-reperfusion injury, vasodilation, carcinogenesis or inhibition of cancer, anti-inflammatory and regulation of hormone metabolism [9]. An increasing amount of evidences indicate that H_2_S regulates autophagy in many diseases, such as ischemia/reperfusion injury [10], lung disease [11] and neurodegenerative disease [12]. In this review, we summarize recent studies on the role of H_2_S in the pathological processes of the nervous system by regulating autophagy, and analyze the mechanism in detail, hoping to provide references for future related research.

## 2. Overview of Autophagy

**Figure 1:**
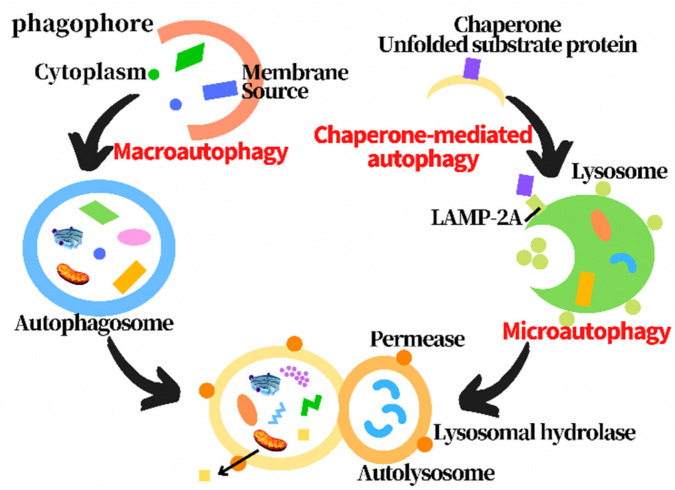
Diagram of three types of autophagy processes.

Autophagy is a catabolic process through which cellular components, including proteins, lipids and organelles, are degraded in lysosomes and recycled to promote cellular homeostasis [13]. In the process of autophagy, the abnormal organelles and proteins, and pathogens are wrapped in autophagosomes formed by double membranes, and then transferred to lysosomes to be degraded [14]. Three types of autophagy have been found, including macroautophagy, microautophagy and chaperone-mediated autophagy, according to the substrate selectivity and the delivery path of the cargo to the lysosome cavity [15,16]. Macroautophagy, commonly known as autophagy, is the most thoroughly studied one, including initiation, expansion, closure and degradation processes [17]. Macroautophagy firstly forms cytosolic double membrane vesicles called autophagosomes to isolate the cargo. The autophagosomes then fuse with lysosomes to form autophagolysosomes, and the cargo is then degraded by the protease [18]. Chaperone-mediated autophagy transports a single unfolded and labeled protein directly across the lysosomal membrane. Microautophagy involves the direct uptake of cargo through lysosomal membrane invagination. All three types of autophagy lead to the degradation of cargo and transport the decomposition products back to the cytoplasm for cell reuse (Figure 1) [14,19,20,21,22,23,24,25]. Under physiological conditions, autophagy is usually at a basic level. Activated by various cellular stresses, including nutrient/energy starvation, endoplasmic reticulum stress, hypoxia, hypoxia, and organelle damage, the enhanced autophagy can clear the abnormal proteins in cells to maintain cell survival [26]. However, if autophagy is maintained at a high level for a long time due to internal and external factors, it may lead to autophagic death of cells to cause diseases. Therefore, the effect of autophagy on cells is a “double-edged sword” [27]. Autophagy disorders can be involved in a variety of pathological processes, including type 2 diabetes and obesity, infectious diseases and inflammation, neurodegenerative diseases and cancers [28]. In the pathological processes, the abnormal autophagy lost the function of clearing abnormal substances in the cell, leading to autophagic death [29]. However, the mechanism is not completely clear. 

## 3. Overview of H_2_S

**Figure 2:**
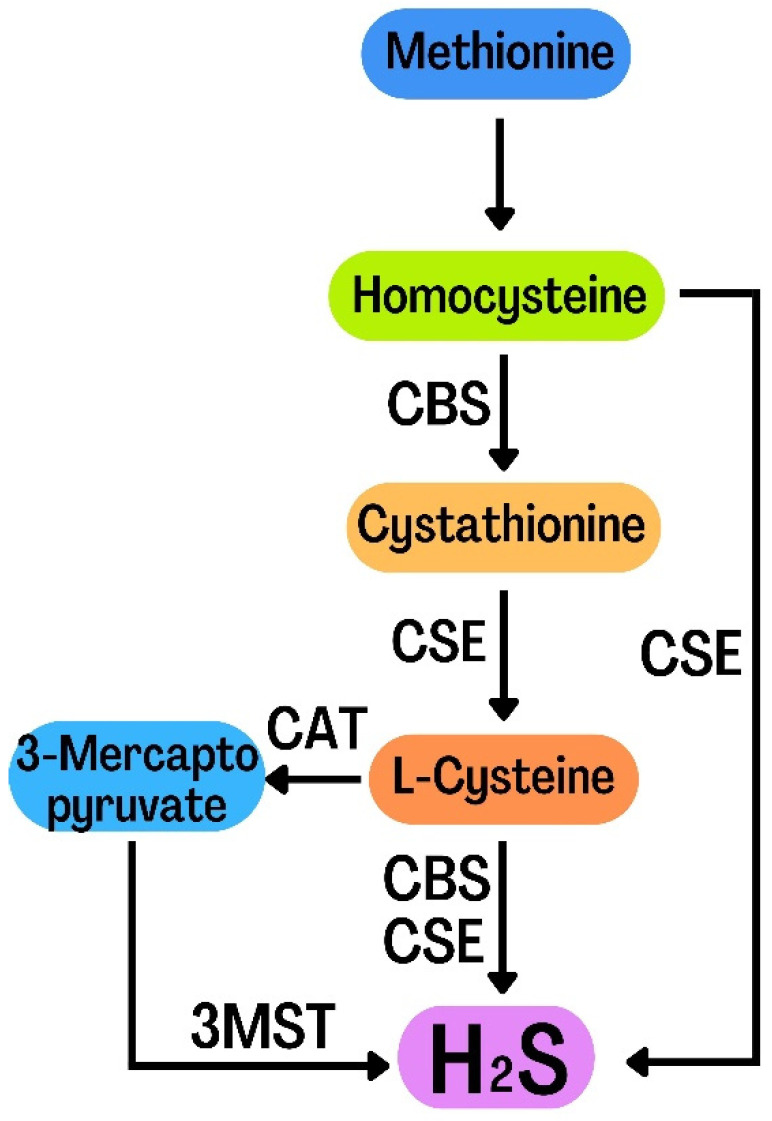
Diagram of the endogenous H_2_S generation process.

H_2_S is a colorless, flammable, lipophilic molecule with an unpleasant smell, similar to rotten eggs [30]. For many years, H_2_S has been simply regarded as a toxic gas and environmental pollutant [31]. Abe and Kimura’s 1996 report proposed the role of endogenous H_2_S in neural regulation, ushering in a new era of H_2_S study and its role in biology [32]. The evidence shows that H_2_S can regulate the function of the nervous system, especially the hippocampus. H_2_S not only plays an important regulatory role in the nervous system, but also plays an important role in digestive, cardiovascular system, urinary and blood systems. Therefore, H_2_S has been considered as the third gas signal molecule after CO and NO [33]. Endogenous H_2_S is mainly produced under the catalysis of three enzymes:cystathionine-β-synthase(CBS), cystathionine-γ-lyase (CSE) and 3-mercaptopyruvate sulfurtransferase (3-MST)[34]. During the production of endogenous H_2_S, L-homocysteine (Hcy) is converted into cystathionine, which is then converted into L-cysteine. CBS and CSE catalyse L-cysteine to produce H_2_S. L-cysteine was catalyzed by CAT to generate 3-mercaptopyruvate (3-MP) and eventually H_2_S. Moreover, 3-MST catalyzes 3-MP to produce H_2_S. Hcy is also catalyzed by CSE to produce H_2_S (Figure 2) [35,36]. H_2_S has many physiological functions, such as anti-apoptosis, anti-inflammatory, anti-oxidative stress, vasodilation and lowering blood pressure [37]. The biological function of H_2_S is mainly achieved by reversible protein vulcanization [38]. H_2_S mainly plays its physiological function by regulating cell function. There are many mechanisms for its regulation of cell function: histone modification, DNA methylation, DNA damage repair and H_2_S post-translational modification of proteins through sulfur hydration [39]. In recent years, the evidence has shown that H_2_S plays an important role by regulating autophagy in the pathological processes of the nervous system, including traumatic brain injury, nervous system hypoxia-ischemia injury, sleep deprivation-induced cognitive impairment, diabetic depression and Parkinson’s disease. However, the relevant mechanisms have not been fully understood. In this review, we summarize the literature on the role of H_2_S in regulating autophagy in the pathological processes of the nervous system, and analyze the related mechanism, in order to provide a reference for future research.

## 4. H_2_S Plays a Protective Role by Regulating Autophagy in Traumatic Brain Injury

Traumatic brain injury (TBI) refers to the interruption of brain function or other pathological changes of the brain caused by external forces. It is estimated that the annual incidence rate of TBI in the world is 50 million cases, and TBI is the major cause of the disability and death worldwide [40,41,42]. The secondary injury (subsequent biochemical changes) of TBI can lead to cell death, such as autophagic cell death and apoptosis, resulting in neurological impairment. Therefore, the inhibition of secondary cell death is the focus of brain injury treatment [43,44]. Mingyang Zhang and colleagues found that exogenous H_2_S ameliorated TBI of mice by decreasing brain edema, improving movement disorder and spatial memory acquisition after brain injury. Mechanism research revealed that H_2_S decreased the acute plasmalemma permeability in injured cells of the cortical and hippocampal brain regions in mice with TBI. The plasma membrane permeability is a marker of apoptosis and autophagy. Therefore, the subsequent detection of apoptosis and autophagy showed that H_2_S abolished TBI-induced cleaved caspase-3 and decline of Bcl-2, inhibited LC3-II, Beclin-1 and Vps34 activation and reversed the decline of p62 in the cortex and hippocampus of mice with TBI, indicating that H_2_S suppressed apoptosis and autophagy in TBI model of mice [45]. It has been reported that autophagic death and apoptosis participate in TBI [46]. Hence, in the above study, it can be deduced that exogenous H_2_S improves TBI of mice through inhibiting autophagy and apoptosis, which needs to be further confirmed [45]. In addition, Beclin 1 interacts with Bcl-2 through its BH3 domain [47], suggesting that autophagy and apoptosis can regulate each other. In the above, H_2_S reverses the upregulation of the Beclin-1/Bcl-2 ratio induced by TBI, which indicated that H_2_S inhibits apoptosis and autophagy through regulating Beclin-1-Vps34 interaction.

Mitochondria is a dynamic and multifunctional organelle, which plays an important role in maintaining the balance of intracellular environment and the function and survival of cells [48,49]. It has been reported that protecting mitochondria is important for TBI [50,51,52]. The results of Kebin Xu et al. showed that exogenous H_2_S preserved the integrity of blood–brain barrier (BBB) by increasing the expression of adherens junctions (AJs) and tight junctions (TJs), ameliorating pericyte survival, and mitigating neurovascular defect. H_2_S also protected neurons from apoptosis through decreasing apoptotic cells number and increasing Bcl-2/Bax ratio. Moreover, H2S induced remyelination and axonal repair through stabilizing microtubules and mitigating mitochondrial dysfunction. In addition, H_2_S suppressed autophagy following TBI, which was caused by the activation of the PI3K/AKT/mTOR pathway. Rapamycin (an autophagy activator) reversed H_2_S protection of TBI, while 3-MA (an autophagy suppressor) had the opposite effect, indicating that H_2_S improved TBI by inhibiting autophagy. Collectively, exogenous H_2_S ameliorated TBI through suppressing autophagy via activating PI3K/AKT/mTOR pathway [53]. The study showed that autophagy can promote cell survival through eliminating the damaged organelles and protein [27]. In H_2_S improvement of TBI, autophagy promotes cell death after TBI via the excessive degradation of basic cellular components, which is inhibited by H_2_S.

In addition to exogenous H_2_S, the endogenous H_2_S may also improve TBI. 3-MST is an important enzyme regulating endogenous H_2_S synthesis [54,55]. To explore 3-MST changes after TBI and its possible role, Mingyang Zhang et al. established a mouse model of TBI through a controlled cortical impingement system. The results showed that 3-MST existed in the cerebral cortex of normal mice. It increased gradually to reach a peak on the first day after TBI, and then dropped to a valley on the third day. Moreover, 3-MST collocated with neuron. Additionally, autophagy also peaked evidenced by the increased expression of LC3 on the first day after TBI. Moreover, the TBI-induced 3-MST was partially labeled by LC3. This indicated that some of the neurons expressing 3-MST, not dying neurons, were LC3 positive. However, 3-MST was not collocated with propidium iodide (cell death marker), and LC3 positive cells were partially colocalized with propidium iodide, suggesting that a considerable proportion of dead cells underwent autophagic cell death, and 3-MST has a protective effect on brain injury [56]. The evidence indicates that autophagy can maintain the survival of nerve cells [57,58]. Therefore, in the above study, it can be deduced that the TBI-induced 3-MST in cerebral cortex is related to the autophagic protection of neurons after TBI, suggesting that endogenous H_2_S may play an important role in autophagic cell death after TBI [56]. Whether endogenous H_2_S can improve TBI through autophagy needs further research.

## 5. H_2_S Plays a Protective Role by Regulating Autophagy in Nervous System Hypoxia-Ischemia Injury

### 5.1. H_2_S Plays a Protective Role by Regulating Autophagy in Spinal Cord Ischemia-Reperfusion Injury

Spinal cord ischemia-reperfusion (I/R) injury is a dynamic process and one of the most devastating complications during thoracic-abdominal aortic surgery, which can lead to the severe nerve defect of lower limbs and even brain death [59,60]. However, the pathological mechanism of spinal cord I/R injury is not completely clear, and there is no effective neuroprotective therapy [61]. It has been reported that autophagy is involved in spinal cord I/R injury; however, whether autophagy plays a protective or harmful role in spinal cord I/R injury is still uncertain [62,63]. Lei Li and colleagues established an in vivo and in vitro spinal cord I/R injury model and conducted a series of experiments. The results showed that exogenous H_2_S decreased the infarcted area of spinal cord and ameliorated the motor function of hind limbs of a rat model of spinal cord I/R injury. Mechanism research showed that H_2_S treatment decreased miR-30c expression and induced autophagy by upregulating the expression of Beclin-1 and LC3II in spinal cord of rat with spinal cord I/R injury. The results in OGD-induced spinal cord I/R injury of SY-SH-5Y cells were similar to those in vivo. Moreover, miR-30c negatively regulated Beclin-1 expression by targeting its 3′UTR, indicating that miR-30c negatively regulated autophagy in spinal cord with I/R injury. Similarly, exogenous H_2_S also suppressed Beclin-1 3′UTR in SY-SH-5Y cells with Oxygen, Glucose Deprivation (OGD)-induced spinal cord I/R injury. In addition, pretreatment of 3-MA or pre-miR-30c abolished H_2_S improvement of spinal cord I/R injury, indicating that exogenous H_2_S improved spinal cord I/R injury by promoting autophagy through inhibiting miR-30c [64]. The study indicates that in the case of extensive mitochondrial damage, autophagy can clear the damaged mitochondria to protect cells before it releases death-inducing proteins [63,65]. In the early stage of I/R injury, autophagy is upregulated to protect cells [66]. Therefore, in the above study, it can be deduced that H_2_S can improve spinal I/R injury by promoting autophagy and clearing the damaged mitochondria caused by I/R injury. Furthermore, it has been reported that the inhibition of autophagy improves spinal cord I/R injury [62], which is contrary to the above conclusion. The reason may be the different periods of spinal I/R injury, which needs to be studied further.

### 5.2. H_2_S Plays a Protective Role by Regulating Autoophagy in the Hypoxia-Ischemia Brain Injury of Neonatal Mice

Perinatal brain injury induced by hypoxia-ischemia (HI) may lead to neurodevelopmental disorders. Improving perinatal care can greatly improve the survival of infants with brain injury. In the critical period of brain development, HI can lead to perinatal brain neuron excitotoxicity, brain cell apoptosis and microglia activation [67,68]. H_2_S has been reported to play neuroprotective role in the central nervous system [69,70]. However, whether H_2_S can improve HI brain injury through regulating autophagy is not clear. Danqing Xin et al. found that L-Cysteine treatment after HI decreased early brain injury and improved behavioral deficits and synaptic damage in neonatal mice, which is related to the increased expression of synaptophysin and postsynaptic density protein 95 expression in the damaged cortex. In-depth research showed that L-cysteine could reduce the aggregation of CD11b^+^/CD45^high^ cells, inhibit the activation of microglia and astrocytes, and decrease the upregulation of reactive oxygen species (ROS), malondialdehyde, neuronal apoptosis and inflammatory gene expression induced by HI in the damaged cortex of neonatal mice. Furthermore, L-Cysteine promoted autophagy by upregulating the expression of LC3 II and Beclin1 and downregulating p62 expression in the injured cortex after HI. CQ, an inhibitor of autophagy, abolished the protective effect of L-Cysteine on HI brain injury, indicating that L-Cysteine improved HI brain injury by promoting autophagy. In addition, the treatment of amino-oxyacetic acid (a suppressor of the H_2_S-producing enzyme) reversed the protective effect of L-Cysteine on HI brain injury. Collectively, endogenous H_2_S produced by L-Cysteine ameliorated HI-induced brain injury of neonatal mice by promoting autophagy [71]. In the above study, the enhanced autophagy can reduce ROS-mediated cell injury by scavenging HI-induced damaged mitochondria. Previous studies have shown that exogenous H2S promotes autophagy through the mTOR pathway [11,72,73]. Furthermore, Stat3 pathway has been reported to be involved in autophagy [74]. L-Cysteine inhibited mTOR and Stat3 pathway, suggesting that the endogenous H_2_S produced by L-Cysteine might promote autophagy by inhibiting the mTOR and Stat3 pathway, which needs to be further confirmed [71].

## 6. H_2_S Plays a Protective Role by Regulating Autophagy in Sleep Deprivation-Induced Cognitive Impairment

Sleep is very important to maintain the balance of physiological internal environment. Therefore, insufficient sleep will lead to various diseases. Sleep deprivation (SD) is an increasingly serious health problem in contemporary society [75]. SD can lead to cognitive impairment; however, the mechanism is not completely clear [76,77]. H_2_S has been reported to improve cognitive impairment [78]; however, how H_2_S inhibits SD-induced cognitive impairment has not been thoroughly studied. The results of Shan Gao and colleagues showed that exogenous H_2_S alleviated SD-induced cognitive impairment by ameliorating working memory impairment in Y-maze test, cognitive dysfunction in the novel object recognition test, location memory deficit in object location test, and spatial learning and memory disorder in the Morris water maze test, which were reversed by the inhibition of Sirt-1 by Sirtinol (an inhibitor of Sirit-1). H_2_S also reduced SD-induced hippocampal excessive autophagy by decreasing autophagosomes, downregulating Beclin1, and upregulating p62 in the hippocampus of SD-exposed rats. Furthermore, Sirtinol reversed H_2_S inhibition of the cognitive impairment and excessive hippocampal autophagy induced by SD in rats [79]. In addition, it has been reported that H_2_S increased Sirt-1 expression in the hippocampus of SD-exposed rats [80]. Collectively, it can be deduced that exogenous H_2_S mitigates SD-induced cognitive impairment by inhibiting autophagy via hippocampal Sirt-1 of rats [79]. Another study demonstrated that hippocampal excessive autophagy and inhibition of endogenous H_2_S production results in SD-induced cognitive impairment [81], which further confirmed the protective effect of H_2_S on SD-induced cognitive impairment.

## 7. H_2_S Plays a Protective Role by Regulating Autophagy in Diabetic Depression

The incidence rate of depression in people with diabetes is higher than that in people without diabetes [82,83]. Therefore, it is particularly important to study the mechanism of depression in diabetes. Brain-derived neurotropic factor (BDNF) has been reported to play an important role in depression [84,85]; however, the mechanism is not completely clear. Hai Yao Liu and colleagues found that exogenous H_2_S activated BDNF-TrkB pathway by increasing the protein expressions of BDNF and p-TrkB in the hippocampus of streptozotocin (STZ)-induced diabetic rats. K252a, which is an inhibitor of BDNF-TrkB pathway, abolished the antidepressant effect of H_2_S as evidenced by the tail suspension, novelty suppressed feeding, forced swimming and elevated plus-maze tests. Moreover, K252a reversed H_2_S-promoted hippocampal autophagy by downregulating the protein expression level of Beclin-1 and upregulating the protein expression of p62 in diabetic rats. Summarily, exogenous H2S improved depression by promoting autophagy via activating the BDNF-TrkB pathway [86]. In the above studies, in addition to BDNF-TrkB pathway, whether H_2_S can regulate autophagy through other ways to play an antidepressant role remains to be studied. In addition, the evidence indicates that the injury of hippocampal neurons contributes to diabetic depression [87,88]. Hence, future studies are needed to clarify whether H_2_S inhibits hippocampal neuronal damage by regulating autophagy.

## 8. H_2_S Plays a Protective Role by Regulating Autophagy in Parkinson’s Disease

Parkinson’s disease (PD) is a common progressive neurodegenerative disorder, the prevalence of which rises with advancing age. It affects about 2% of the population worldwide [89,90,91]. The neuronal apoptosis in the substantia has been reported to contribute to PD [92,93]. Wu Jiang et al. found that exogenous H_2_S mitigated neuronal apoptosis in the substantia by inhibiting 6-hydroxydopamine (OHDA)-induced TUNEL-positive cells, caspase-3 activity and Bax expression and mitigated 6-OHDA-induced reduction of Bcl-2 expression in substantia nigra of rats. In-depth research showed that 6-OHDA upregulated the expressions of Beclin-1, LC3-II and P62, increased the autophagosomes number and decreased the autolysosomes number in the substantia nigra, which were reversed by NaHS treatment, indicating that exogenous H_2_S restored the autophagy flux of substantia nigra impaired by 6-OHDA in rats. Moreover, H_2_S abolished 6-OHDA-induced decrease of leptin expression in the substantia nigra, and leptin-OBR, an inhibitor of leptin signaling, mitigated H_2_S inhibition of neuronal apoptosis and H_2_S promotion of the impaired autophagy in substantia nigra of rats treated by 6-OHDA. Summarily, exogenous H_2_S ameliorated neuronal apoptosis in substantia nigra by promoting autophagy impaired by 6-OHDA via activating leptin signaling in PD, which needed to be further confirmed by using the autophagy inhibitor [94]. It has been reported that mitochondrial dysfunction plays an important role in PD [95,96]. In the above study, exogenous H_2_S upregulates Bcl-2 expression and downregulates Bax expression, suggesting that H_2_S may inhibit mitochondrial-mediated neuronal apoptosis, which need to be studied further. Evidence indicates that the leptin signaling promotes autophagy [97,98]. The in-depth mechanism of H_2_S alleviating neuronal apoptosis through upregulating autophagy via leptin remains to be clarified.

## 9. Conclusions

In this review, we summarize the recent studies about the role of H_2_S targeting autophagy in the pathological processes of the nervous system as follows: (1) exogenous H_2_S ameliorates TBI of mice through suppressing autophagy and apoptosis; (2) exogenous H_2_S improves TBI by inhibiting autophagy via activating PI3K/AKT/mTOR pathway; (3) endogenous H_2_S may play protective role against TBI by inhibiting autophagic cell death; (4) exogenous H_2_S ameliorates spinal I/R injury through promoting autophagy and clearing the damaged mitochondria caused by I/R injury; (5) endogenous H_2_S produced by L-Cysteine improves HI-induced brain injury of neonatal mice by promoting autophagy via inhibiting mTOR and Stat3 pathway; (6) exogenous H_2_S alleviates SD-induced cognitive impairment by inhibiting autophagy via hippocampal Sirt-1; (7) exogenous H_2_S ameliorates depression through promoting autophagy by activating BDNF-TrkB pathway; (8) exogenous H_2_S improves neuronal apoptosis in substantia nigra through promoting autophagy impaired by 6-OHDA via activating leptin signaling (Table 1). It can be seen from the above that H_2_S sometimes promotes autophagy, and sometimes inhibits autophagy to protect the nervous system. The reason may be related to the types of nervous system diseases and the different course of nervous system disease. Generally speaking, at the beginning of the pathological process, the enhanced autophagy can help cells adapt to in vitro and in vivo stimulation and promote intracellular homeostasis, while the continuously enhanced autophagy can lead to autophagic death, thereby aggravating the pathological process. In addition, H_2_S regulates autophagy in the nervous system through a variety of pathways, including PI3K/AKT/mTOR pathway, mTOR/Stat3 pathway, Sirt-1 pathway, BDNF-TrkB pathway and leptin pathway. Whether there are other pathways to participate remains to be clarified in future research.

Our previous studies have shown that exogenous H_2_S can target autophagy/NLRP3 inflammasome and play a protective role in the liver [72,99]. Therefore, whether H_2_S can improve nervous system diseases by regulating autophagy/NLRP3 inflammasome is a topic worthy of study in the future.

With the deepening of relevant studies, H_2_S inhibition of autophagy death of neural cells may become a new therapeutic strategy for the treatment of neurological diseases.

## Figures and Tables

**Table 1:** The summary of the role of H_2_S targeting autophagy in the pathological processes of the nervous system.

The Type of Nervous System Diseases	The Role of H2S Targeting Autophagy	Experimental Model	Reference
Traumatic brain injury (TBI)	Exogenous H_2_S ameliorates TBI of mice through suppressing autophagy and apoptosis	Mice model of TBI	[45]
TBI	Exogenous H_2_S improves TBI by inhibiting autophagy via activating PI3K/AKT/mTOR pathway	Mice/SH-SY5Y cells model of TBI	[53]
TBI	Endogenous H_2_S may play protective role against TBI by inhibiting autophagic cell death	Mice model of TBI	[56]
Spinal cord ischemia-reperfusion (I/R) injury	Exogenous H_2_S ameliorates spinal I/R injury through promoting autophagy and clearing the damaged mitochondria caused by I/R injury	Rat model of spinal cord ischemia-reperfusion injury	[64]
hypoxia-ischemia (HI) brain injury	Endogenous H_2_S produced by L-Cysteine improves HI-induced brain injury of neonatal mice by promoting autophagy via inhibiting the mTOR and Stat3 pathway	Neonatal mice model of hypoxia-ischemia injury	[71]
sleep deprivation (SD)-induced cognitive impairment	Exogenous H_2_S alleviates SD-induced cognitive impairment by inhibiting autophagy via hippocampal Sirt-1	Mice model of SD-induced cognitive impairment	[79]
diabetes depression	Exogenous H_2_S ameliorates depression through promoting autophagy by activating BDNF-TrkB pathway	Rat model of diabetes depression	[86]
Parkinson’s disease (PD)	Exogenous H_2_S improves neuronal apoptosis in substantia nigra through promoting autophagy impaired by 6-OHDA via activating leptin signaling	6-hydroxydopamine rat model of PD	[94]
